# Filgotinib in Moderate-to-Severe Crohn’s Disease: A Network Meta-Analysis of Efficacy and Adverse Events

**DOI:** 10.3390/healthcare14010005

**Published:** 2025-12-19

**Authors:** Yasser Ali Khoshaim, Yahya Z. Habis, Afnan Ghazi Daqnah, Razan Khalid Alqurashi, Yazeed Shaker Abdulrahim, Abdullah Sakkat, Sultan Ali Alsubhi, Deema Tawfeq Almuwlad, Halah Samer Bukhari, Abdulrhman J. Shogdar, Omar Ashraf Amir, Mohamed Sayed Zaazouee

**Affiliations:** 1 Department of Internal Medicine, King Abdullah Medical Complex, Jeddah 23816, Saudi Arabia; khoshaimy85@gmail.com; 2 Department of Internal Medicine, Faculty of Medicine, King Abdulaziz University, Jeddah 21589, Saudi Arabia; yhabis@kau.edu.sa (Y.Z.H.); asakkat@kau.edu.sa (A.S.); 3 Department of Internal Medicine, King Abdulaziz University Hospital, Jeddah 21534, Saudi Arabia; 4 East Jeddah General Hospital, Jeddah 22253, Saudi Arabia; afnandegnah@gmail.com (A.G.D.); yazeed.s.s.a@gmail.com (Y.S.A.); 5 Home Health Care Center, Jeddah First Health Cluster, Jeddah 22233, Saudi Arabia; dr.razan.aq@gmail.com; 6 Faculty of Medicine, King Abdulaziz University, Rabigh 21589, Saudi Arabia; exoticc1002@gmail.com (S.A.A.); shogdar1@gmail.com (A.J.S.); 7 Faculty of Medicine, Umm Al-Qura University, Mecca 24381, Saudi Arabia; oideema76@gmail.com (D.T.A.); h259006@gmail.com (H.S.B.); 8 Home Health Care, King Abdullah Medical Complex, Jeddah 23816, Saudi Arabia; omaramirr95@gmail.com; 9 Faculty of Medicine, Al-Azhar University, Assiut 71524, Egypt

**Keywords:** filgotinib, JAK1 inhibitor, Crohn’s disease, systematic review, network meta-analysis

## Abstract

**Background:** Filgotinib is an emerging Janus kinase 1 (JAK1) inhibitor being investigated for inflammatory bowel disease. This systematic review and network meta-analysis (NMA) evaluated the efficacy and safety of filgotinib in adult patients with moderate-to-severe crohn’s disease. **Methods:** We systematically searched PubMed, EMBASE, and Scopus through April 2025. Randomized controlled trials evaluating filgotinib versus placebo in adults with moderate-to-severe Crohn’s disease were included. Primary outcomes were clinical remission and endoscopic response. Study quality was assessed using the Cochrane Risk of Bias 2.0 tool. A network meta-analysis was performed to integrate direct and indirect evidence, reporting risk ratios (RRs) with 95% confidence intervals (CIs). **Results:** Five randomized controlled trials (from 4 publications) met the inclusion criteria. Filgotinib 200 mg significantly improved clinical remission compared with placebo (RR: 1.75 [1.40–2.19]) and 100 mg (RR: 1.38 [1.11–1.71]), while 100 mg showed no significant difference versus placebo (RR: 1.27 [0.99–1.63]). For endoscopic response, both 200 mg (RR: 1.72 [1.09–2.69]) and 100 mg (RR: 1.65 [1.02–2.69]) demonstrated significant benefit over placebo, though no difference was observed between active doses (RR: 1.04 [0.64–1.68]; I^2^ = 57%). In the two-item patient-reported outcome, 200 mg showed significant improvement versus placebo (RR: 1.47 [1.20–1.80]) and 100 mg (RR: 1.26 [1.02–1.55]), while 100 mg remained insignificant versus placebo (RR: 1.17 [0.93–1.46]). Neither dose increased the risk of treatment-emergent adverse events, serious adverse events, or infections compared with placebo, with consistent homogeneity across analyses. **Conclusions:** Filgotinib 200 mg demonstrated superior efficacy across clinical, endoscopic, and patient-reported outcomes compared with 100 mg and placebo, with a favorable safety profile. The 100 mg dose showed limited efficacy and no advantage over placebo. Filgotinib represents a promising oral therapeutic option, particularly for biologic-naïve patients and in maintenance therapy, while also showing potential benefit in perianal fistulising crohn’s disease. Future trials should explore long-term safety and head-to-head comparisons with established biologics.

## 1. Introduction

Crohn’s disease (CD) represents a chronic inflammatory disorder of the gastrointestinal tract characterized by transmural inflammation, often presenting with abdominal pain, diarrhea, weight loss, and extraintestinal manifestations [1]. With a global prevalence of 3–20 cases per 100,000 individuals and rising incidence rates particularly in Western populations, CD imposes substantial disease burden, diminished quality of life, and increased healthcare expenditures [2]. Despite significant advancements in therapeutic approaches over the past two decades, approximately 30–40% of patients demonstrate primary non-response to current biological therapies, with an additional 30–50% experiencing secondary loss of response over time, underscoring the critical need for novel therapeutic agents with distinct mechanisms of action [3,4].

Janus kinase (JAK) inhibitors have emerged as a promising therapeutic class in immune-mediated inflammatory disorders through their capacity to modulate multiple cytokine signaling pathways simultaneously [5]. Filgotinib, a selective JAK1 inhibitor, has demonstrated particular interest due to its preferential inhibition profile that potentially enhanced efficacy while minimizing adverse events commonly associated with pan-JAK inhibition [6]. The oral administration route of filgotinib presents a noteworthy advantage over currently available parenteral biological therapies, potentially improving treatment adherence and patient satisfaction [7].

Recent clinical trials investigating filgotinib in moderate-to-severe CD have reported encouraging outcomes regarding clinical remission, endoscopic response, and mucosal healing parameters. The FITZROY phase II trial provided initial evidence of efficacy with a favorable short-term safety profile [8]. Subsequent phase III investigations within the DIVERSITY clinical program have expanded the evidence base regarding filgotinib’s therapeutic potential in both induction and maintenance settings across various CD patient populations, including those with prior anti-TNF exposure or failure [9].

Previous conventional meta-analysis suggested that filgotinib 200 mg provides superior short-term clinical remission and mucosal healing compared with placebo, while the 100 mg dose shows limited efficacy, with both doses maintaining acceptable safety profiles [10]. However, those analyses were restricted to pairwise comparisons and did not formally assess the relative efficacy between doses.

This network meta-analysis (NMA) aims to evaluate the comparative efficacy and safety of filgotinib 100 mg and 200 mg versus placebo in moderate-to-severe CD. By integrating both direct and indirect evidence, we assessed clinical remission rates, patient-reported outcomes, endoscopic response, and adverse events, thereby providing a comprehensive and dose-specific evaluation of filgotinib’s therapeutic profile.

## 2. Materials and Methods

This Frequentist NMA was conducted in accordance with the guidelines of the Cochrane Handbook for Systematic Reviews of Interventions and reported according to the Preferred Reporting Items for Systematic Reviews and Meta-Analyses (PRISMA) statement [11,12]. The protocol was registered on OSF (https://doi.org/10.17605/OSF.IO/WT7DK).

### 2.1. Eligibility Criteria

We included randomized controlled trials (RCTs) that evaluated at least one of the predefined outcomes related to clinical remission, endoscopic response, patient-reported outcomes, or adverse events of filgotinib in adult patients (≥18 years) with moderate-to-severe CD. We excluded non-randomized trials, observational studies, reviews, letters, conference abstracts, and ongoing or unpublished trials without sufficient data. Participants were required to have a clinical diagnosis of CD, with no restrictions placed on prior treatment exposure, disease duration, or sex.

Eligible interventions included filgotinib administered orally at a dose of either 100 mg or 200 mg once daily, compared to placebo. The primary efficacy outcomes were clinical remission, defined as a CD Activity Index (CDAI) score of less than 150, and endoscopic response, defined as a reduction of at least 50% in the Simple Endoscopic Score for CD (SES-CD). Secondary efficacy outcomes included improvement in the two-item patient-reported outcome (PRO2), a composite score based on daily stool frequency and self-reported abdominal pain, with remission defined as “7 × (mean daily number of liquid or very soft stools) + 7 × (mean daily self-reported abdominal pain)”. Safety outcomes included the incidence of treatment-emergent adverse events (TEAEs), serious TEAEs, and any infection.

### 2.2. Information Sources, Search Strategy, and Study Selection

We searched PubMed, EMBASE, and Scopus from database inception until April 2025, without language or publication date restrictions. We also manually screened the reference lists of included studies and relevant review articles to identify additional eligible trials. The search strategy combined MeSH terms and keywords related to filgotinib and CD. Full search strategies for each database are provided in Table S1. All identified records were imported into EndNote software 21.3, and duplicates were removed. Four reviewers independently screened titles and abstracts to assess eligibility. Full-text articles of potentially relevant studies were retrieved and assessed for inclusion. Discrepancies were resolved by consensus or consultation with a fifth reviewer.

### 2.3. Data Collection Process

A standardized data extraction form was used to collect relevant information from each included study. Three reviewers independently extracted data on study characteristics, participant demographics, intervention and comparator details, outcome definitions and results, follow-up duration, baseline data, and outcomes. Discrepancies between reviewers were resolved through discussion.

### 2.4. Risk of Bias Assessment

The methodological quality of each included study was assessed using the Cochrane Risk of Bias 2.0 (RoB 2) tool [13]. Three reviewers independently evaluated the risk of bias across five domains: the randomization process, deviations from intended interventions, missing outcome data, measurement of the outcome, and selection of the reported result. Each domain was rated as low risk of bias, some concerns, or high risk of bias. Any disagreements were resolved through consensus.

### 2.5. Statistical Analysis

A network meta-analysis was performed to compare filgotinib 100 mg, 200 mg, and placebo by synthesizing both direct and indirect evidence using R programming. The analysis was conducted within a frequentist statistical framework. For dichotomous outcomes, we calculated pooled risk ratios (RRs) with 95% confidence intervals (CIs). A random-effects model and leave-one-out test were used to account for expected clinical and methodological heterogeneity across studies. Heterogeneity was assessed using the Chi-squared test and quantified using the I^2^ statistic, with a *p* value less than 0.10 considered indicative of substantial heterogeneity. Publication bias and small-study effects were assessed using comparison-adjusted funnel plots and Egger’s regression test for each outcome.

The assumption of transitivity was evaluated by ensuring that the included trials were sufficiently similar in terms of patient characteristics, interventions, and outcome definitions to justify indirect comparisons within the network framework.

### 2.6. Certainty of Evidence

The certainty of evidence for each outcome was assessed using the GRADE (Grading of Recommendations, Assessment, Development and Evaluation) approach [14]. The assessment considered study design, risk of bias, inconsistency, indirectness, imprecision, and potential publication bias. Randomized controlled trials (RCTs) were initially rated as high-quality evidence and subsequently downgraded based on the applicable domains. The overall quality of evidence for each outcome was categorized as high, moderate, low, or very low.

## 3. Results

**Figure 1:**
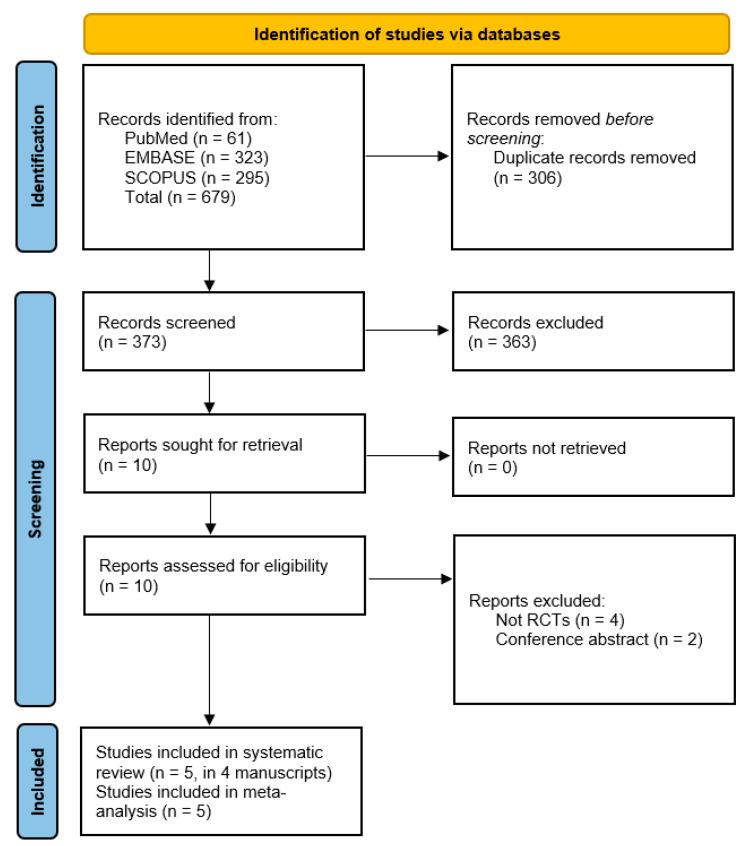
PRISMA flow diagram of study selection.

Out of 679 records identified from PubMed, EMBASE, and Scopus, 306 duplicates were removed. After screening 373 records, 363 were excluded. Of the 10 reports assessed for eligibility, 6 were excluded because they were not RCTs or conference abstracts. Finally, 5 studies (in 4 manuscripts) were included in both the systematic review and meta-analysis [8,9,15,16], Figure 1.

### 3.1. Baseline Characteristics and Summary of the Included Studies

The average age ranged from 35.1 to 46 years, with female representation varying from 43% to 71.9%. Disease duration averaged between 6.8 and 14.6 years, indicating a chronic condition. Baseline CDAI scores ranged from 190 to 309, while SES-CD scores were reported in some studies, indicating endoscopic disease activity. Most participants had a history of previous interventions, including anti-TNF agents and immunomodulators, and were on concomitant medications such as oral steroids and antibiotics. Further details are shown in Table 1 and Table 2.

### 3.2. Quality Assessment

**Figure 2:**
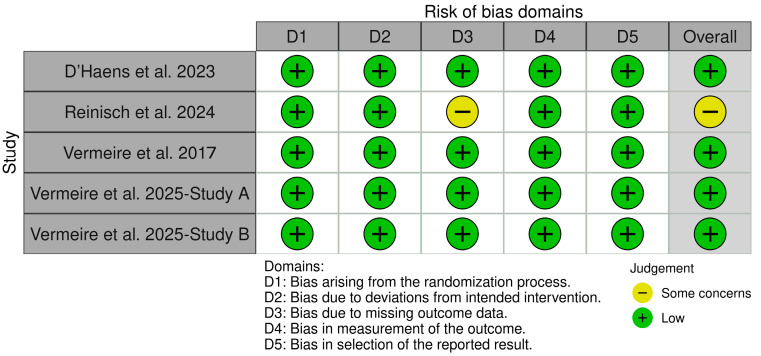
Risk of bias assessment of included studies using the ROB 2 tool [8,9,15,16].

Using the ROB 2 tool, the quality assessment of the included studies revealed low risk of bias in most domains. Vermeire et al. (2017), Vermeire et al. (2025) (study A and Study B), and D’Haens et al. (2023) were rated as low risk across all five domains, indicating high methodological quality [8,9,16]. Reinisch et al. (2024) showed “some concerns” in missing outcomes data and the overall risk of bias, despite being rated low in the other domains [15], Figure 2.

### 3.3. Outcomes

#### 3.3.1. Efficacy Outcomes

##### Clinical Remission CDAI < 150 Points

**Figure 3:**
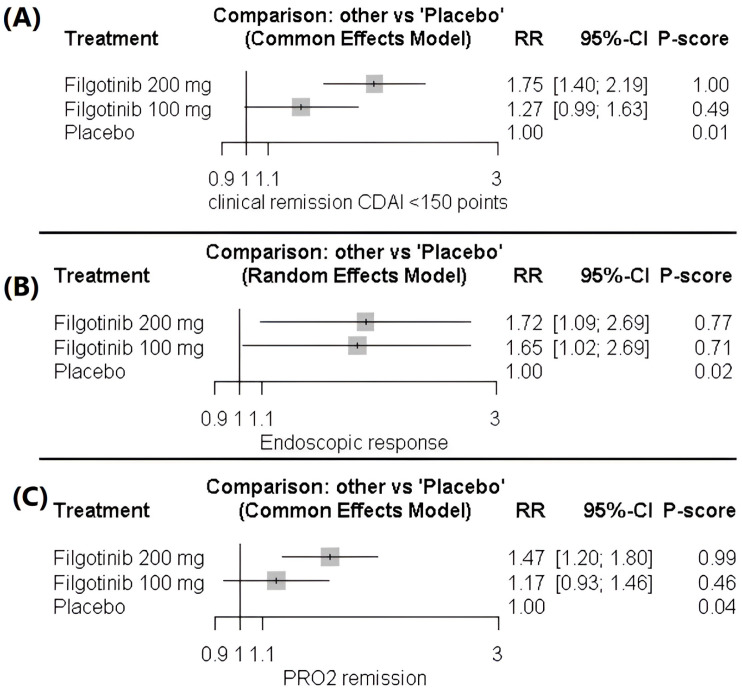
(**A**) Forest plot of clinical remission (CDAI < 150). (**B**) Forest plot of endoscopic response (≥50% reduction in SES-CD). (**C**) Forest plot of improvement in two-item patient-reported outcome (PRO2).

Filgotinib 200 mg demonstrated a significant improvement in the clinical remission rate compared with both 100 mg (1.38 [1.11–1.71]) and placebo (1.75 [1.40–2.19]). In contrast, filgotinib 100 mg did not differ significantly from placebo (1.27 [0.99–1.63]). The analysis indicated homogeneity across studies (*p* = 0.88, I^2^ = 0), Figure 3A, Table S2 and Figure S1.

##### Endoscopic Response (Reduction of at Least 50% in Centrally Read SES-CD)

For the endoscopic response outcome, both filgotinib 200 mg (1.72 [1.09–2.69]) and 100 mg (1.65 [1.02–2.69]) were associated with significant improvements compared with placebo. In contrast, the two active doses did not differ significantly (1.04 [0.64–1.68]), and the data were heterogeneous (*p* = 0.072, I^2^ = 57%), Figure 3B, Table S3 and Figure S2.

Heterogeneity was resolved after excluding Vermeire et al. (2025) [9]—Study B, reducing inconsistency to non-significant levels (*p* = 0.53, I^2^ = 0). Following this adjustment, Filgotinib 200 mg demonstrated an endoscopic response of RR 2.32 (95% CI 1.66–3.24), and Filgotinib 100 mg showed RR 2.06 (95% CI 1.44–2.97), both compared with placebo. There remained no significant difference between the 200 mg and 100 mg doses after adjustment, with an RR of 0.89 (95% CI 0.60–1.31), Table S4, and Figures S3 and S4.

##### Two-Item Patient-Reported Outcome (PRO2)

Filgotinib 200 mg demonstrated a significant improvement in the two-item patient-reported outcome (PRO2) compared with both filgotinib 100 mg (1.26 [1.02–1.55]) and placebo (1.47 [1.20–1.80]). In contrast, filgotinib 100 mg did not differ significantly from placebo (1.17 [0.93–1.46]). The data showed homogeneity (*p* = 0.3, I^2^ = 16.5%), Figure 3C, Table S5 and Figure S5.

#### 3.3.2. Safety Outcomes

##### Treatment-Emergent Adverse Events (TEAEs)

**Figure 4:**
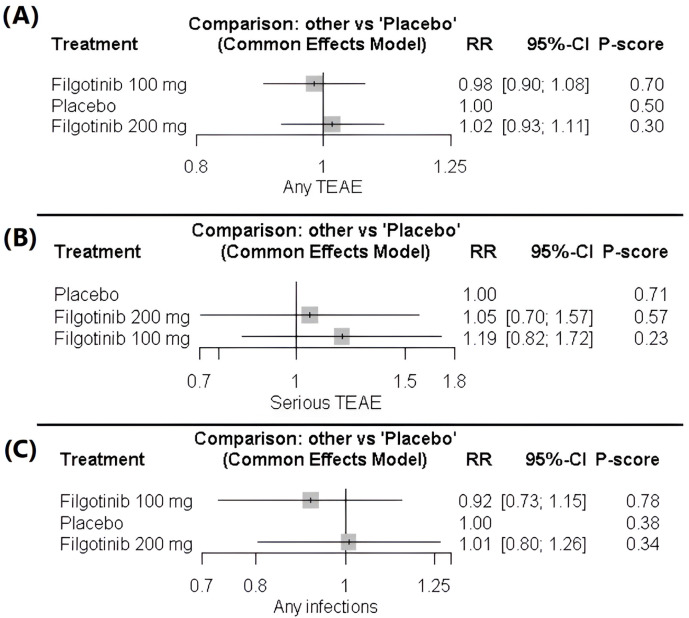
(**A**) Forest plot of treatment-emergent adverse events (TEAEs). (**B**) Forest plot of serious treatment-emergent adverse events (TEAEs). (**C**) Forest plot of infection risk.

No significant differences were observed in the risk of any TEAE with filgotinib 200 mg (1.02 [0.93; 1.11]) or 100 mg (0.98 [0.90; 1.08]) compared with placebo. Likewise, the two active doses did not differ significantly (1.03 [0.95; 1.12]). The findings were consistent across studies, indicating homogeneity. The data were homogeneous (*p* = 0.54, I^2^ = 0), Figure 4A, Table S6 and Figure S6.

##### Serious Treatment-Emergent Adverse Events

The analysis of serious TEAEs did not differ significantly across treatment groups. Filgotinib 200 mg (1.05 [0.70–1.57]) and filgotinib 100 mg (1.19 [0.82–1.72]) were insignificant compared with placebo, and the difference between the two active doses was also insignificant (0.89 [0.60–1.31]). The data showed homogeneity (*p* = 0.14, I^2^ = 37.6%), Figure 4B, Table S7 and Figure S7.

##### Occurrence of Any Infection

No significant differences were observed in the risk of any infection with filgotinib 200 mg (1.01 [0.80–1.26]) or 100 mg (0.92 [0.73–1.15]) compared with placebo. Similarly, there was no significant difference between the two doses (1.10 [0.87–1.39]). The data indicated homogeneity across studies (*p* = 0.717, I^2^ = 0), Figure 4C, Table S8 and Figure S8.

#### 3.3.3. Qualitative Synthesis

##### Role of Filgotinib in Perianal Fistulizing CD (PFCD)

Filgotinib has shown promise in treating PFCD as demonstrated in the DIVERGENCE 2 trial [15]. This phase 2 study revealed that filgotinib 200 mg led to significant reductions in the number of draining perianal fistulas compared to placebo, with a combined fistula response rate of 47.1%. The treatment was generally well tolerated, highlighting its potential as a therapeutic option for patients with PFCD who have experienced prior treatment failures.

##### Difference Between Prior Treatments and Naive Patients

In the DIVERSITY trial, biologic-naive patients exhibited different responses to filgotinib compared to biologic-experienced patients [9]. Biologic-naive patients showed a nominally significant improvement in clinical remission rates with filgotinib 200 mg, while biologic-experienced patients had higher rates of prior treatment failures. This suggests that treatment history may influence the efficacy of filgotinib, indicating a need for tailored therapeutic strategies based on patient history.

##### Maintenance Role of Filgotinib

The maintenance phase of the DIVERSITY study demonstrated that patients who achieved clinical remission or an endoscopic response at week 10 with filgotinib 200 mg continued to benefit from treatment [9]. At week 58, significantly more patients maintained PRO2 clinical remission and endoscopic response compared to placebo. This underscores the importance of ongoing therapy in sustaining remission and highlights filgotinib’s potential as a long-term treatment option for CD.

### 3.4. GRADE Assessment

Efficacy outcomes showed moderate-quality evidence, with the 200 mg dose significantly improving clinical remission and PRO2 scores, while the 100 mg dose did not. The endoscopic response also had moderate-quality evidence, but moderate heterogeneity (I^2^ = 57%). Safety outcomes were supported by high-quality evidence, showing no significant increase in adverse events or infection risk compared to placebo, Table 3.

### 3.5. Publication Bias

Across all outcomes, the comparison-adjusted funnel plots are generally symmetric, and Egger’s tests are non-significant. This indicates no evidence of small-study effects or publication bias, with any observed scatter reflecting imprecision rather than true asymmetry, Figures S9–S14.

## 4. Discussion

Our network meta-analysis demonstrated a clear dose-dependent efficacy profile for filgotinib in moderate-to-severe CD. The 200 mg dose significantly improved clinical remission based on CDAI compared with both placebo and 100 mg, while the 100 mg dose did not differ from placebo. This superiority of 200 mg extended to patient-reported outcomes (PRO2), whereas the 100 mg dose again showed limited efficacy, consistent with the DIVERGENCE 2 trial [9]. The superior efficacy of filgotinib 200 mg likely reflects dose-dependent JAK1 inhibition, with higher doses more effectively suppressing key pro-inflammatory cytokines (e.g., IL-6, IL-10, interferons) and achieving greater modulation of immune activation and mucosal inflammation than the subtherapeutic 100 mg dose [10].

For endoscopic response outcomes, both filgotinib doses (200 mg and 100 mg) were associated with significant improvements compared with placebo, while no significant difference was observed between the two doses. However, this comparison showed moderate heterogeneity. Importantly, filgotinib maintained a favorable safety profile across both dosages. The NMA confirmed no significant differences between either dose and placebo for treatment-emergent adverse events, serious adverse events, or infections, with consistent homogeneity across studies.

The differential efficacy between biologic-naïve and biologic-experienced populations represents another crucial finding. Based on qualitative synthesis, biologic-naïve patients demonstrate a better response to filgotinib, particularly at the 200 mg dose [9]. This pattern aligns with established observations in inflammatory bowel disease therapeutics, where treatment-naïve patients typically exhibit higher response rates than those with previous treatment failures [17]. The underlying mechanisms may involve less immunological complexity, lower disease burden, or absence of anti-drug antibodies from previous biological therapies [18]. The maintenance efficacy demonstrated in the DIVERSITY trial further supports filgotinib’s potential role in long-term disease management [9]. Sustained PRO2 clinical remission and endoscopic response at week 58 indicate durable efficacy, addressing a critical unmet need given the significant proportion of patients who experience secondary loss of response to current biological therapies.

The previous systematic review and meta-analysis by Elgendy et al. highlighted the dose-dependent efficacy of filgotinib, with 200 mg achieving significant improvements in clinical remission and mucosal healing compared with placebo, while 100 mg showed limited benefit, and both doses demonstrated acceptable safety [10]. Building on this foundation, our NMA provides an important extension by synthesizing both direct and indirect evidence, enabling head-to-head comparisons between 100 mg and 200 mg. This allowed us to demonstrate the superior efficacy of 200 mg across clinical, patient-reported, and endoscopic outcomes compared with 100 mg, while confirming comparable safety profiles across doses.

The differential efficacy between 100 mg and 200 mg doses provides valuable clinical guidance for optimal dosing strategies. Based on our results, the 200 mg dose should be considered the preferred option for most patients, particularly those with more severe disease or previous treatment failures. However, individual benefit-risk assessments remain essential, especially in populations with elevated baseline risk factors for JAK inhibitor-associated adverse events.

The maintenance efficacy demonstrated in the DIVERSITY trial suggests filgotinib could address the significant problem of secondary loss of response to biological therapies. Approximately 30–50% of patients experience diminished efficacy over time with current biologics, necessitating dose escalation, switching within class, or exploring alternative mechanisms of action [19]. Filgotinib’s distinct mechanism potentially offers a valuable option for these patients, potentially delaying or preventing surgical interventions. The encouraging results in PFCD from the DIVERGENCE 2 trial merit particular attention [15]. PFCD represents one of the most challenging manifestations of Crohn’s disease, with limited effective medical therapies available [20]. From a mechanistic perspective, these findings reinforce the central role of JAK-STAT signaling in Crohn’s disease pathophysiology. The efficacy of selective JAK1 inhibition highlights the importance of cytokines that signal through this pathway, including IL-6, IL-10, type I and II interferons, and several other inflammatory mediators implicated in disease pathogenesis [5].

The positioning of filgotinib within the evolving Crohn’s disease treatment algorithm warrants careful consideration. Traditional treatment paradigms have followed a stepwise approach from conventional immunomodulators to biological therapies, typically anti-TNF agents as first-line biologics [21]. However, the emergence of oral targeted synthetic small molecules with favorable efficacy-safety profiles challenges this conventional approach. Given its oral administration route, lack of immunogenicity, rapid onset of action, and efficacy across various disease manifestations, including fistulizing disease, filgotinib may offer advantages as an earlier treatment option rather than being reserved for anti-TNF failures. This represents a potential paradigm shift in treatment algorithms, particularly for patients with contraindications or preferences against injectable therapies.

The beneficial effects observed in both clinical and endoscopic outcomes suggest that filgotinib addresses both symptomatic improvement and underlying mucosal inflammation, addressing both patient-reported symptoms and objective disease activity markers. This comprehensive approach to disease control aligns with emerging treat-to-target strategies in inflammatory bowel disease management that emphasize the importance of achieving deep remission to modify the long-term disease course [22].

A key strength of our analysis lies in its inclusion of high-quality randomized controlled trials with consistent outcome definitions, enabling clinically meaningful conclusions. By assessing both induction and maintenance phases, we offer insights into filgotinib’s potential role throughout the disease course. The inclusion of both symptomatic and objective endpoints—clinical response, remission, and endoscopic improvement—enhances the overall relevance of our findings. Furthermore, our systematic evaluation of safety outcomes across several domains, including treatment-emergent adverse events, serious adverse events, and infections, provides detailed safety data to support clinical decision-making. The low heterogeneity observed in most outcomes adds to the strength and reliability of the evidence. Importantly, by employing a network meta-analysis, we were able to integrate both direct and indirect comparisons, allowing a more robust evaluation of dose-dependent effects between 100 mg and 200 mg. However, several limitations warrant acknowledgment. First, the relatively small number of included studies limits the strength of our conclusions, particularly for subgroup analyses. Second, the variable follow-up periods across studies (ranging from 10 to 58 weeks) challenge the assessment of long-term efficacy and safety. Additional long-term extension data will be crucial to evaluate filgotinib’s sustained benefit-risk profile (e.g., malignancy and thromboembolism). The studies included predominantly white populations from Western countries, which may limit their generalizability to other ethnicities and regions with different environmental exposures and genetic backgrounds. Additionally, the trials excluded patients with certain comorbidities, which limits their applicability to more complex real-world populations.

## 5. Conclusions

Our NMA demonstrates that filgotinib 200 mg offers superior efficacy over both placebo and filgotinib 100 mg in achieving clinical remission, patient-reported outcomes, and endoscopic response in moderate-to-severe CD, while maintaining a favorable safety profile. The 100 mg dose provided limited or insignificant benefits compared with placebo, confirming a clear dose–response relationship. Importantly, both doses were well tolerated, with no significant increase in adverse events, serious adverse events, or infections.

Based on these findings, filgotinib 200 mg should be considered the preferred dose for most patients, particularly those who are biologic-naïve or have more severe disease activity. Clinicians should individualize treatment decisions, weighing potential risks in patients with elevated susceptibility to JAK inhibitor–related adverse events. Important research gaps remain regarding filgotinib’s long-term safety, real-world effectiveness, and comparative efficacy against established biologics such as anti-TNF and anti-IL-23 agents. Future large-scale, head-to-head, and longitudinal studies should clarify its therapeutic role, including in perianal fistulizing Crohn’s disease and potential combination strategies.

## Figures and Tables

**Table 1:** Baseline characteristics of the included studies.

Study	Treatment Arm	Age, Years, Mean (SD)	Gender (Female %)	Duration of Disease, Years, Mean (SD)	CDAI Score, Mean (SD)	SES-CD Score, Mean (SD)
D’Haens et al. 2023 [16]	Placebo (n = 18)	45 (12.9)	50	11.2 (9.1)	300 (63.7)	NR
	Filgotinib 100 mg (n = 32)	42 (12.9)	71.9	14.6 (13.7)	297 (64.9)	NR
	Filgotinib 200 mg (n = 28)	46 (16.3)	67.9	10.6 (8.4)	309 (55.7)	NR
Reinisch et al. 2024 [15]	Placebo (n = 15)	39 (11.8)	26.7	7.5 (7.9) (fistula)	190 (57.9)	NR
	Filgotinib 100 mg (n = 25)	41 (14.0)	40	11.9 (11.1) (fistula)	194 (67.1)	NR
	Filgotinib 200 mg (n = 17)	39 (11.2)	52.9	10.3 (8.3) (fistula)	190 (62.4)	NR
Vermeire et al. 2017 [8]	Placebo (n = 44)	35.1 (11.8)	59	6.8 (5.7)	298.6 (56.8)	15.8 (7.2)
	Filgotinib 200 mg (n = 130)	37.4 (11.6)	55	8.8 (8.5)	291.3 (53.8)	14.2 (6.8)
Vermeire et al. 2025 [9] (Induction Study A)	Placebo (n = 237)	38 (14)	55	9.3 (8.4)	320 (59.4)	13 (7.2)
	Filgotinib 100 mg (n = 245)	39 (14.1)	43	9.9 (10.0)	322 (55.5)	14 (7.9)
	Filgotinib 200 mg (n = 222)	39 (13.8)	50	9.2 (8.4)	323 (55.6)	13 (7.1)
Vermeire et al. 2025 [9] (Induction Study B)	Placebo (n = 229)	39 (12.5)	50	13.0 (9.5)	322 (57.5)	15 (7.8)
	Filgotinib 100 mg (n = 228)	42 (13.5)	56	13.3 (9.7)	321 (55.7)	15 (8.2)
	Filgotinib 200 mg (n = 202)	39 (14.2)	56	11.5 (8.0)	306 (54.0)	15 (7.9)

CDAI: Crohn’s Disease Activity Index, SES-CD: Simple Endoscopic Score for Crohn’s Disease, NR: Not Reported, SD: Standard Deviation.

**Table 2:** Summary of included studies.

ID	Study Design	NCT	Patient Details	Previous Interventions	Filgotinib Doses and Duration	Primary Outcomes	Follow-Up	Conclusion
D’Haens et al. 2023 [16]	Multicenter, double-blinded, RCT	NCT03046056	Patients aged 18–75 years with a confirmed diagnosis of CD for ≥6 months (by imaging, histopathology, or ileoscopy)	Corticosteroids, immunomodulators, TNF inhibitors, vedolizumab, or ustekinumab.	Filgotinib 200 mg or 100 mg once daily for 24 weeks.	Clinical remission (CDAI < 150) at Week 24	24 weeks	This study showed that 24 weeks of filgotinib did not significantly surpass placebo in attaining clinical or MaRIA remission. Nevertheless, biomarker responses indicated a pharmacodynamic effect, and both doses were well tolerated.
Reinisch et al. 2024 [15]	Phase 2, Randomized, Placebo-controlled	NCT03077412	Adults with perianal fistulising Crohn’s disease and prior treatment failure.	Prior treatment failure with TNF inhibitors, antibiotics, or immunomodulators.	Filgotinib 200 mg or 100 mg once daily for 24 weeks.	Combined fistula response at Week 24.	24 weeks	Filgotinib 200 mg resulted in numerical decreases in the number of draining perianal fistulas compared to placebo and was generally well tolerated.
Vermeire et al. 2017 [8]	Phase 2, Randomized, Placebo-controlled	NCT02048618	Patients aged 18–75 years with moderate-to-severe Crohn’s disease confirmed by endoscopy (CDAI) score during screening between 220 and 450 inclusive.	Previous exposure to anti-TNF agents.	Filgotinib 200 mg once daily for 10 weeks, followed by 100 mg or placebo for an additional 10 weeks.	Clinical remission (CDAI < 150) at Week 10.	20 weeks	Filgotinib led to clinical remission in a significantly higher number of patients with active Crohn’s disease compared to placebo, while maintaining an acceptable safety profile.
Vermeire et al. 2025 [9]	Phase 3, Randomized, Placebo-controlled	NCT02914561	Adults aged 18–75 years with moderately to severely active Crohn’s disease (CDAI) score during screening between 220 and 450 inclusive).	Induction Study A includes both biologic-naive and biologic-experienced patients, while Induction Study B focuses exclusively on biologic-experienced patients.	Filgotinib 200 mg or 100 mg once daily for 11 weeks.	PRO2 clinical remission and endoscopic response at Week 10.	58 weeks	Filgotinib 200 mg did not achieve the co-primary endpoints by Week 10, but it proved effective in inducing clinical remission and endoscopic response by Week 58.

**Table 3:** GRADE assessment for study outcomes.

Outcome	No. of Studies (Patients)	Study Design	Risk of Bias	Inconsistency	Indirectness	Imprecision	Publication Bias (Egger Test *p* Value)	Quality of Evidence	Comments
Clinical Remission (CDAI < 150)	4 RCTs	RCTs	Low	None	None	Some	0.94	Moderate	200 mg dose showed significant benefit; 100 mg dose no significant difference vs. placebo
Endoscopic Response (≥50% SES-CD reduction)	3 RCTs	RCTs	Low	Moderate (I^2^ = 57%)	None	Some	Not significant	Moderate	Both doses effective vs. placebo; heterogeneity limits confidence in direct dose comparison
Patient-Reported Outcomes (PRO2)	3 RCTs	RCTs	Low	None	None	Some	Not significant	Moderate	200 mg dose significantly better; 100 mg dose not significantly different from placebo
Treatment-Emergent Adverse Events (TEAEs)	5 RCTs	RCTs	Low	None	None	None	0.36	High	No increase in adverse events for either dose vs. placebo
Serious Adverse Events	4 RCTs	RCTs	Low	None	None	None	0.647	High	No significant differences between active doses and placebo
Infection Risk	4 RCTs	RCTs	Low	None	None	None	0.36	High	No increased infection risk with filgotinib vs. placebo

## Data Availability

No new data were created or analyzed in this study. The data presented in this study will be made available by the authors upon request.

## Supplementary Materials

The following supporting information can be downloaded at: https://www.mdpi.com/article/10.3390/healthcare14010005/s1, Table S1: search strategy for each database (Date 28 April 2025); Table S2: Netleague of clinical remission CDAI < 150 points; Table S3: Netleague of Endoscopic response before resolving heterogeneity; Table S4: Netleague of Endoscopic response after resolving heterogeneity; Table S5: Netleague of PRO2 remission; Table S6: Netleague of any TEAEs; Table S7: Netleague of serious TEAEs; Table S8: Netleague of any infection; Figure S1: Net Graph of clinical remission CDAI < 150 points; Figure S2: Net Graph of Endoscopic Response before resolving heterogeneity; Figure S3: Net Graph of Endoscopic Response after resolving heterogeneity; Figure S4: Comparison Graph of Endoscopic Response after resolving heterogeneity; Figure S5: Net Graph of PRO2 remission; Figure S6: Net Graph of any TEAE; Figure S7: Net Graph of serious TEAE; Figure S8: Net Graph of the occurrence of any infection; Figure S9: funnel plot of clinical remission CDAI < 150 points; Figure S10: funnel plot of Endoscopic response; Figure S11: funnel plot of PRO2 remission; Figure S12: funnel plot of any TEAEs; Figure S13: funnel plot of serious TEAEs; Figure S14: funnel plot of any infection.

## Abbreviations

The following abbreviations are used in this manuscript:

CD	Crohn’s Disease
CDAI	Crohn’s Disease Activity Index
SES-CD	Simple Endoscopic Score for Crohn’s Disease
JAK1	Janus Kinase 1
RR	Risk Ratios
CI	Confidence Intervals
SD	Standard Deviation
NMA	Network Meta-Analysis
PRISMA	The Preferred Reporting Items for Systematic Reviews and Meta-Analyses
RCT	Randomized Controlled Trials
PRO2	Two-Item Patient-Reported Outcome
TEAEs	Treatment-Emergent Adverse Events
PFCD	Perianal Fistulizing Crohn’s Disease
