# Supplementary material for: Filgotinib in Moderate-to-Severe Crohn’s Disease: A Network Meta-Analysis of Efficacy and Adverse Events
Source: Healthcare (Basel). 2025 Dec 19;14(1):5. doi: 10.3390/healthcare14010005 (PMC12785585; doi:10.3390/healthcare14010005)

**Table S1**: search strategy for each database (Date 28 April 2025).

| Database | Search Strategy |
|---|---|
| **PubMed** | ("Filgotinib"[Mesh] OR Filgotinib[tiab] OR GLPG0634[tiab]) AND ("Crohn Disease"[Mesh] OR "Crohn's disease"[tiab] OR "Crohns disease"[tiab] OR "Regional enteritis"[tiab]) |
| **Scopus** | TITLE-ABS-KEY(filgotinib OR GLPG0634) AND TITLE-ABS-KEY("Crohn's disease" OR "Crohns disease" OR "Crohn disease" OR "Regional enteritis") |
| **Embase** | ('filgotinib'/exp OR filgotinib:ti,ab OR glpg0634:ti,ab) AND ('crohn disease'/exp OR 'crohn's disease':ti,ab OR 'crohns disease':ti,ab OR 'regional enteritis':ti,ab) |

**Table S2:** Netleague of clinical remission CDAI <150 points.

| | | |
|---|---|---|
| **Filgotinib 200 mg** | | |
| **1.38 [1.11; 1.71]** | **Filgotinib 100 mg** | |
| **1.75 [1.40; 2.19]** | 1.27 [0.99; 1.63] | **Placebo** |

**Table S3:** Netleague of Endoscopic response before resolving heterogeneity

| | | |
|---|---|---|
| **Filgotinib 200 mg** | | |
| 1.04 [0.64; 1.68] | **Filgotinib 100 mg** | |
| **1.72 [1.09; 2.69]** | **1.65 [1.02; 2.69]** | **Placebo** |

**Table S4:** Netleague of Endoscopic response after resolving heterogeneity

| | | |
|---|---|---|
| **Filgotinib 200 mg** | | |
| 0.89 [0.60; 1.31] | **Filgotinib 100 mg** | |
| **1.05 [0.70; 1.57]** | **1.19 [0.82; 1.72]** | **Placebo** |

**Table S5:** Netleague of PRO2 remission.

| | | |
|---|---|---|
| **Filgotinib 200 mg** | | |
| **1.26 [1.02; 1.55]** | **Filgotinib 100 mg** | |
| **1.47 [1.20; 1.80]** | 1.17 [0.93; 1.46] | **Placebo** |

**Table S6:** Netleague of any TEAEs.

| | | |
|---|---|---|
| **Filgotinib 200 mg** | | |
| 1.03 [0.95; 1.12] | **Filgotinib 100 mg** | |
| 1.02 [0.93; 1.11] | 0.98 [0.90; 1.08] | **Placebo** |

**Table S7:** Netleague of serious TEAEs.

| | | |
|---|---|---|
| **Filgotinib 200 mg** | | |
| 0.89 [0.60; 1.31] | **Filgotinib 100 mg** | |
| 1.05 [0.70; 1.57] | 1.19 [0.82; 1.72] | **Placebo** |

**Table S8:** Netleague of any infection.

| | | |
|---|---|---|
| **Filgotinib 200 mg** | | |
| 1.10 [0.87; 1.39] | **Filgotinib 100 mg** | |
| 1.01 [0.80; 1.26] | 0.92 [0.73; 1.15] | **Placebo** |

**Figure S1:** Net Graph of clinical remission CDAI <150 points.

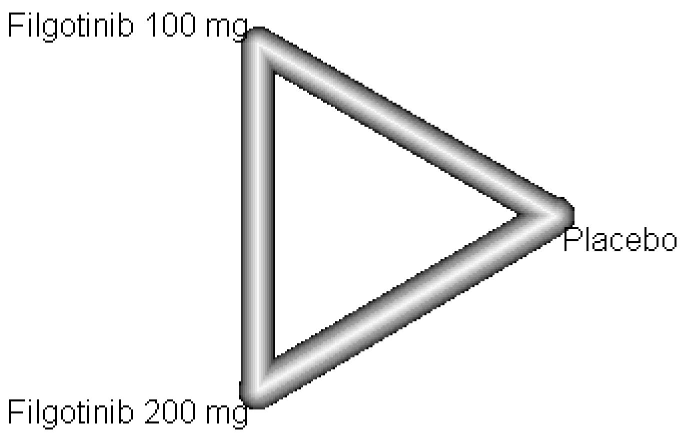


**Figure S2:** Net Graph of Endoscopic Response before resolving heterogeneity

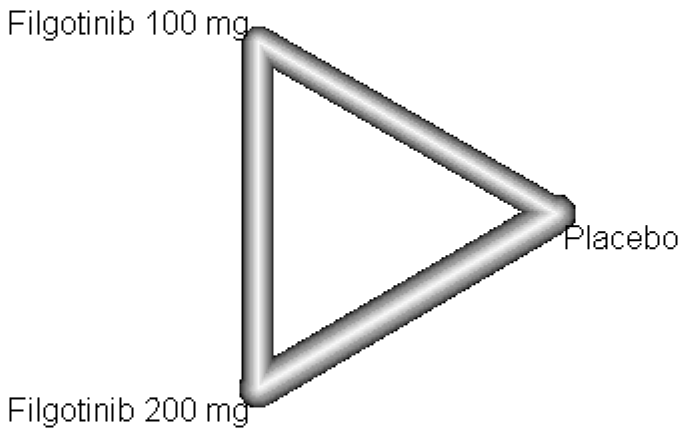


**Figure S3:** Net Graph of Endoscopic Response after resolving heterogeneity

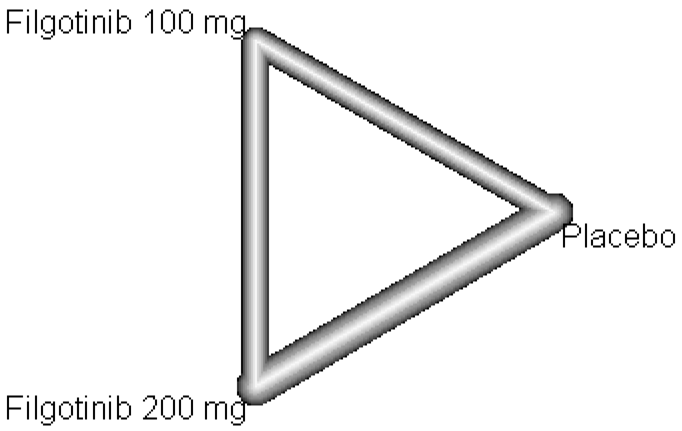


**Figure S4:** Comparison Graph of Endoscopic Response after resolving heterogeneity

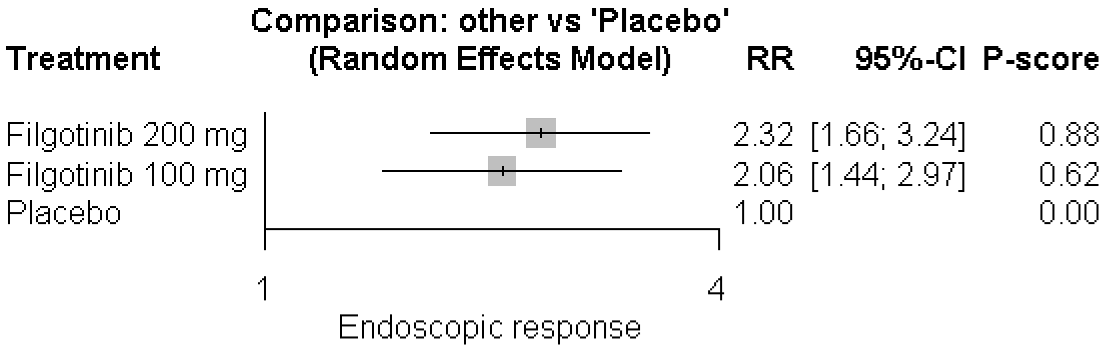


**Figure S5:** Net Graph of PRO2 remission.

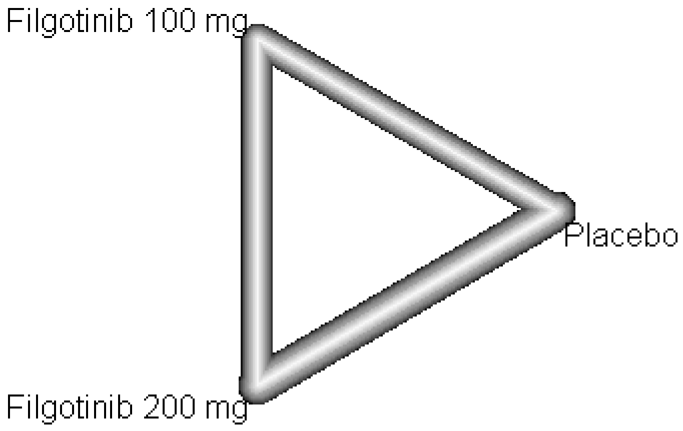


**Figure S6:** Net Graph of any TEAE.

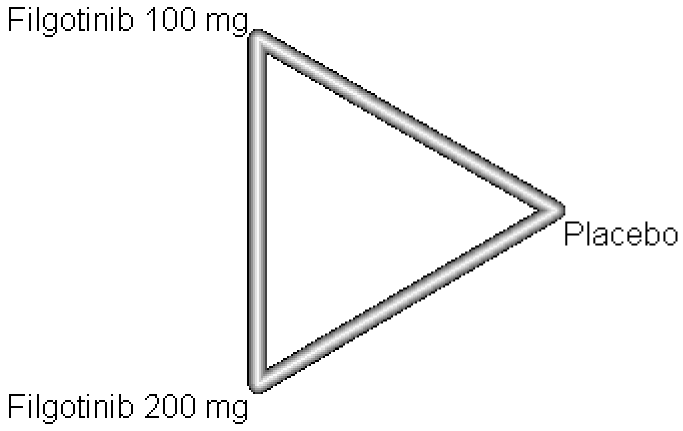

**Figure S7:** Net Graph of serious TEAE.

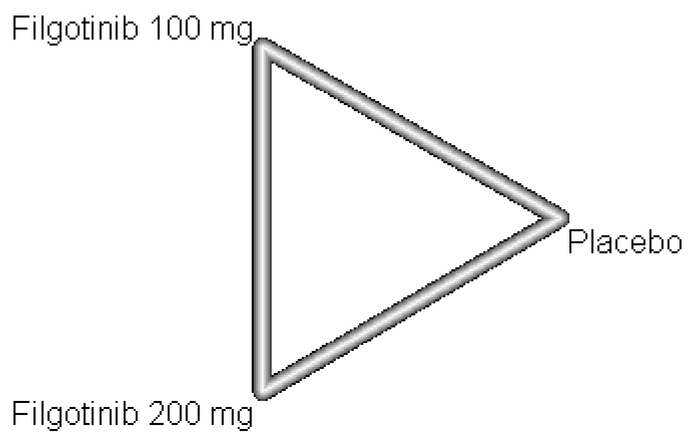


**Figure S8:** Net Graph of the occurrence of any infection.

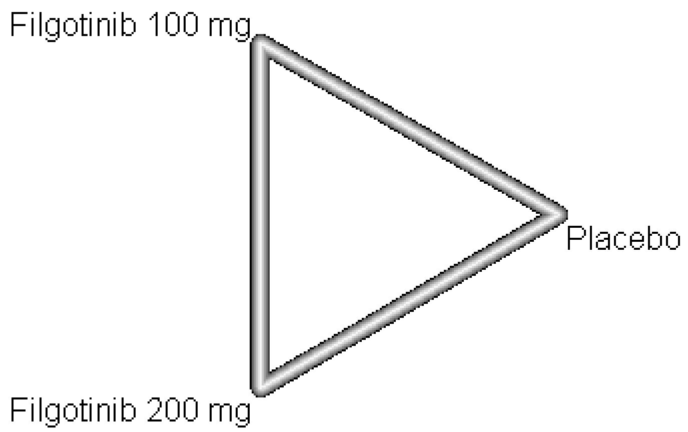

**Figure S9:** funnel plot of clinical remission CDAI <150 points.

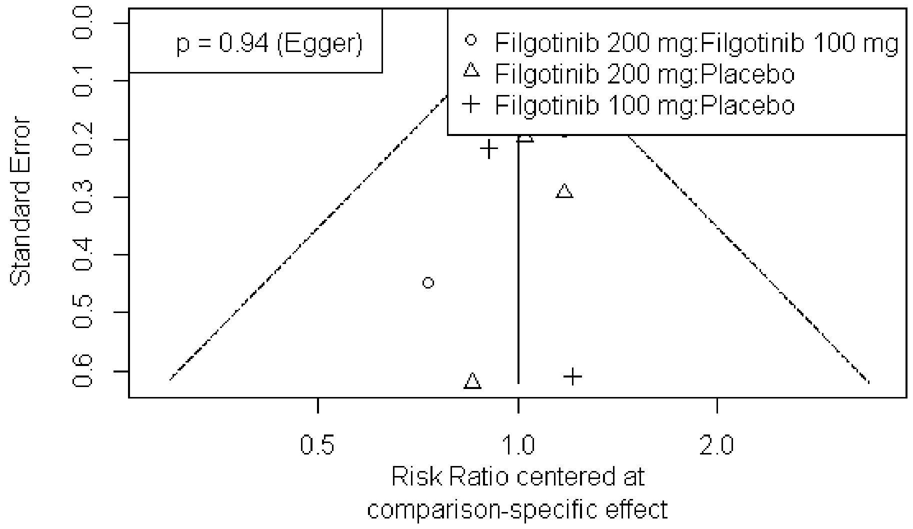


**Figure S10:** funnel plot of Endoscopic response

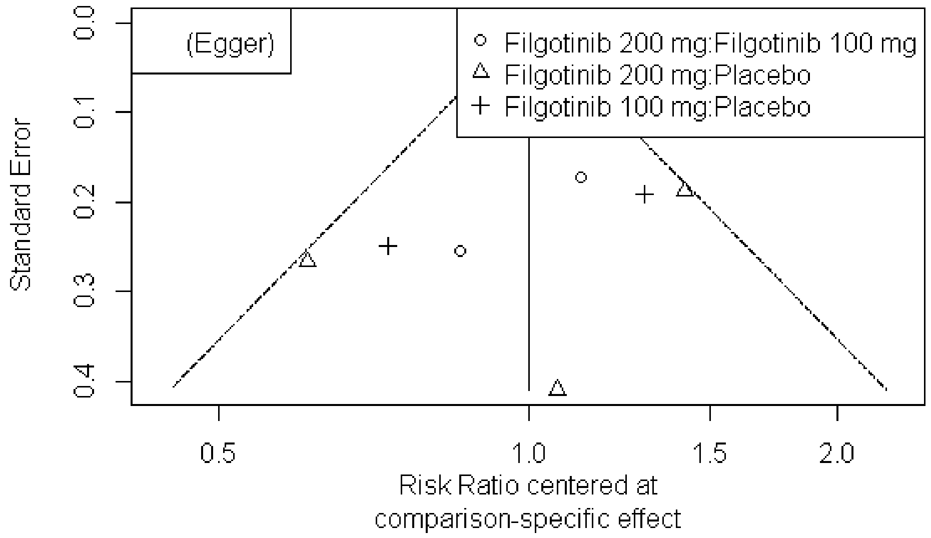


**Figure S11:** funnel plot of PRO2 remission.

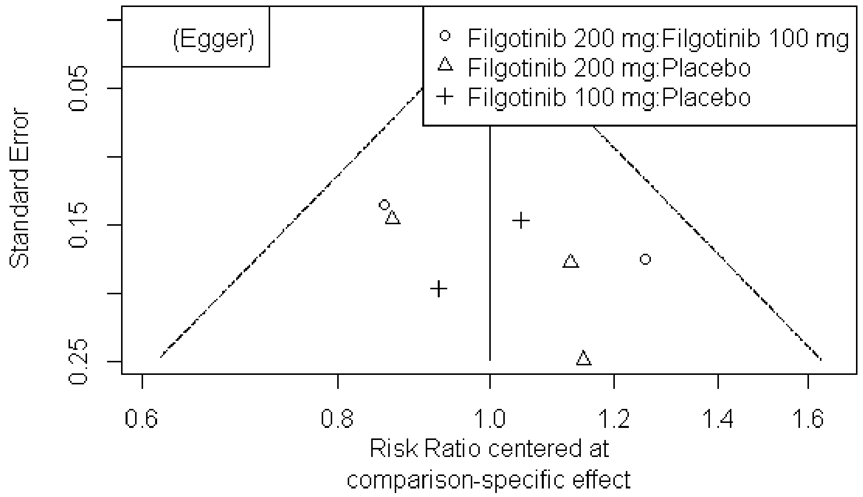


**Figure S12:** funnel plot of any TEAEs.

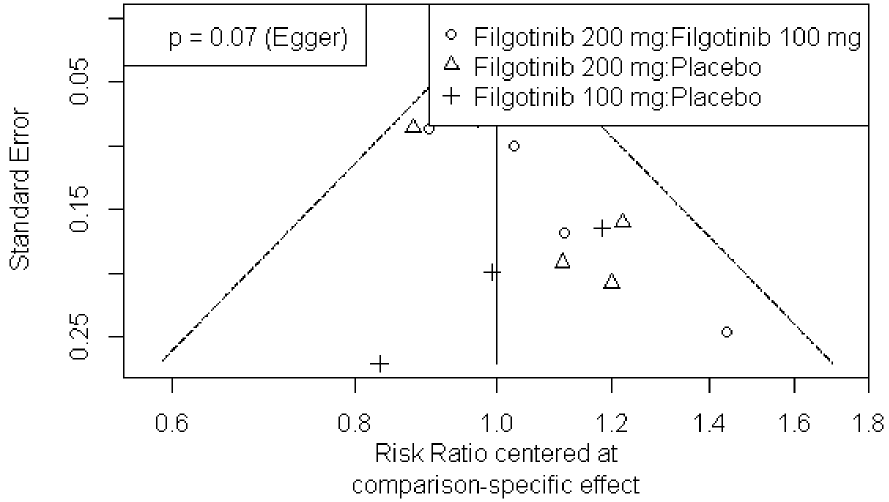


**Figure S13:** funnel plot of serious TEAEs.

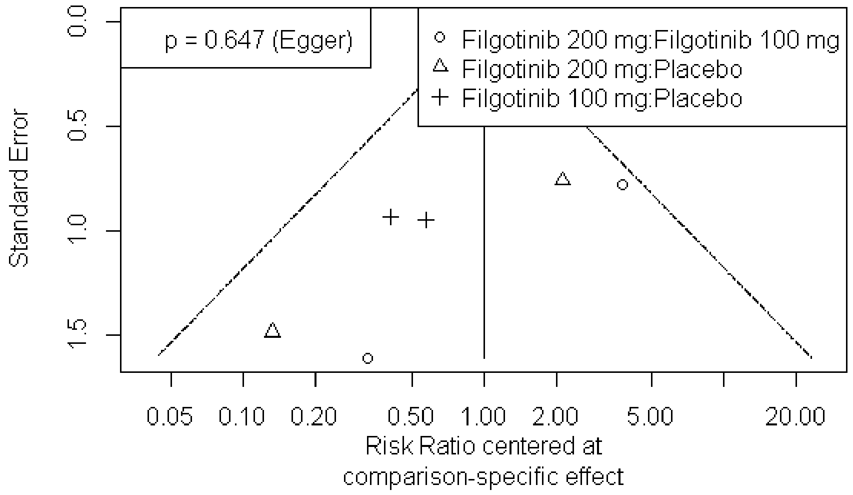


**Figure S14:** funnel plot of any infection.

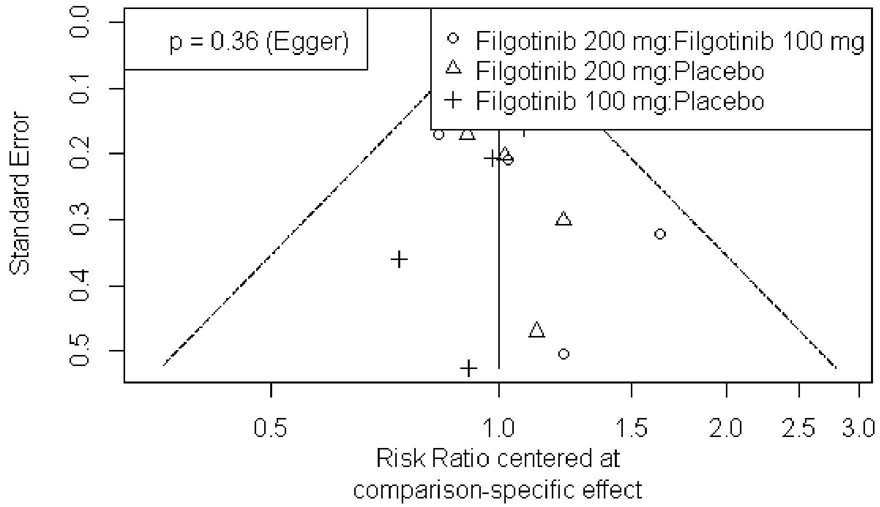

Supplement: Supplementary file 1 [file healthcare-14-00005-s001.zip › healthcare-3913904-supplementary.pdf]
